# Dynamic changes in vasohibin and nitric oxide signaling following surgical resection of head and neck squamous cell carcinoma

**DOI:** 10.1186/s12957-025-03853-8

**Published:** 2025-06-07

**Authors:** Ying-Hsuan Tai, Hsiang-Ling Wu, You-Hsiang Chu, Cheng-Hsien Wu, Shyh-Kuan Tai, Tso-Chou Lin, Shung-Tai Ho, Chih-Cherng Lu

**Affiliations:** 1 Department of Anesthesiology, Shuang Ho Hospital, Taipei Medical University, New Taipei City, 23561 Taiwan; 2 Department of Anesthesiology, School of Medicine, College of Medicine, Taipei Medical University, Taipei, 11031 Taiwan; 3 Department of Anesthesiology, Taipei Veterans General Hospital, Taipei, 11217 Taiwan; 4 School of Medicine, National Yang Ming Chiao Tung University, Taipei, 11221 Taiwan; 5 Graduate Institute of Life Sciences, National Defense Medical Center, Taipei, 11490 Taiwan; 6 Division of Oral and Maxillofacial Surgery, Department of Stomatology, Taipei Veterans General Hospital, Taipei, 11217 Taiwan; 7 Department of Otolaryngology, Taipei Veterans General Hospital, Taipei, 11217 Taiwan; 8 Department of Anesthesiology, Tri-Service General Hospital, National Defense Medical Center, Taipei, 11490 Taiwan; 9 Department of Anesthesiology, Kaohsiung Medical University Hospital, Kaohsiung Medical University, Kaohsiung, 80756 Taiwan; 10 Institute of Aerospace Medicine, National Defense Medical Center, Taipei, 11490 Taiwan

**Keywords:** Angiogenesis, Biomarker, Head and neck cancer, Nitric oxide, Vasohibin

## Abstract

**Background:**

Angiogenesis is essential for tumor growth and metastasis, with various molecules, including vasohibin (VASH), nitric oxide (NO), and inducible nitric oxide synthase (iNOS), implicated in its regulation and potential prognostic value in oncology. However, their roles in modulating surgery-induced angiogenesis in head and neck squamous cell carcinoma (HNSCC) remain unclear. Therefore, the objective of the study was to assess the dynamic changes in VASH, NO, and iNOS levels in HNSCC patients undergoing surgical resection.

**Methods:**

We prospectively enrolled patients with histology-proven HNSCC who underwent surgical resection of primary tumors at the medical center between May and November 2021. Non-cancer controls were recruited to compare baseline biomarker levels with those of HNSCC patients. We measured preoperative and postoperative levels of VASH1 and VASH2 in plasma and leukocytes using enzyme-linked immunosorbent assays and Western blotting, NO using nitrate/nitrite colorimetric assays, and iNOS phosphorylation levels in leukocyte membranes using Western blotting.

**Results:**

Patients with HNSCC (*n* = 15) exhibited elevated baseline levels of VASH1, NO, and leukocyte-induced iNOS phosphorylation compared to non-cancer controls (*n* = 15). After tumor resection, plasma VASH1 levels were significantly downregulated (2233 ± 1464 pg·mL^−1^ vs. 2425 ± 1493 pg·mL^−1^, *p* = 0.0085), while plasma VASH2 levels remained unchanged in HNSCC patients. Similarly, VASH1 levels in leukocytes were reduced after surgery (0.85 ± 0.04 fold,* p* = 0.0068), while VASH2 levels did not change significantly. NO levels in plasma decreased significantly following surgery (0.29 ± 0.09 fold, *p* = 0.0001). Conversely, iNOS phosphorylation levels in leukocytes increased after surgery (1.52 ± 0.10 folds, *p* = 0.0024). The 3-year overall survival rates were 85.7% in patients with lower change folds of VASH1 in leukocytes, compared to 100.0% in those with higher change folds.

**Conclusions:**

This study demonstrated that dynamic changes in VASH and NO signaling following tumor resection could serve as a potential indicator of tumor angiogenesis. Our findings suggest that the overall activity of the VASH pathway in leukocytes was reduced after tumor removal, highlighting the potential of leukocyte physiology as a novel biomarker for cancer surveillance and control.

**Supplementary Information:**

The online version contains supplementary material available at 10.1186/s12957-025-03853-8.

## Introduction

Angiogenesis, the process of forming new blood vessels, is a hallmark of cancer, playing a pivotal role in tumor growth, progression, and metastasis by supplying nutrients and oxygen to malignant cells [1]. Dysregulated angiogenesis disrupts the balance between stimulatory and inhibitory factors, enabling tumors to thrive and spread. Studying angiogenesis is critical in oncology, as targeting this process offers opportunities for novel diagnostics and therapies to halt cancer progression. Key molecules involved in angiogenesis include vasohibin-1 (VASH1) [2–4], vasohibin-2 (VASH2) [5–8], and nitric oxide (NO) [9, 10]. VASH1 and VASH2, as regulators of angiogenesis, exert opposing effects, while NO influences vascular dynamics, collectively shaping the tumor microenvironment [2–10]. Understanding their roles provides a foundation for exploring their potential as biomarkers or therapeutic targets in cancer.

VASH1 and VASH2 play contrasting roles in angiogenesis, a critical process driving tumor growth and metastasis in head and neck squamous cell carcinoma (HNSCC) [11]. VASH1, primarily expressed in endothelial cells (ECs), acts as a negative feedback regulator of angiogenesis, induced by vascular endothelial growth factor receptor (VEGFR) signaling via VEGFR2 and protein kinase C-delta (PKCδ), as well as by fibroblast growth factor [2–4]. In contrast, VASH2, expressed in infiltrating mononuclear cells or cancer cells, promotes angiogenesis and is frequently associated with poorer prognosis across various cancer types [5–8]. Notably, despite its anti-angiogenic role in ECs, elevated VASH1 expression in tumor blood vessels has been associated with poorer oncological prognosis and increased tumor recurrence, suggesting a complex role in tumor progression [12–16]. The interplay between VASH1 and VASH2 underscores their potential as prognostic biomarkers, with VASH1 levels in circulating white blood cells (WBCs) possibly indicating anti-angiogenic capacity, tumor behavior, and response to therapies.

NO, synthesized primarily by inducible nitric oxide synthase (iNOS) in response to inflammatory and pathological stimuli, is a key regulator of diverse physiological and pathological processes, including tumor growth, angiogenesis, and metastasis [9, 10]. iNOS-driven NO production promotes tumor vascularization by enhancing vasodilatation and increasing tumor vasculature permeability, often in concert with pathways like prostaglandin E2 signaling [17]. Our prior research identified stage-dependent changes in plasma levels of angiopoietin-1, angiopoietin-2, and NO following HNSCC tumor resection, indicating their role in an endogenous anti-angiogenic response to malignancy [18]. However, the specific impact of surgical resection on VASH1 and VASH2 concentrations, and the interplay between NO signaling and vasohibins in modulating surgery-induced angiogenesis, remain unexplored in HNSCC.

This study primarily aimed to evaluate the dynamic changes in angiogenesis-regulating molecules, specifically VASH1, VASH2, and NO in plasma, and VASH1, VASH2, and iNOS in circulating WBCs, in HNSCC patients undergoing surgical resection. By measuring these markers before and after surgery, we sought to elucidate their roles in modulating anti-angiogenic responses and their potential as prognostic indicators for survival. Additionally, we investigated the influence of the tumor process on these markers by comparing their baseline levels in plasma (VASH1, VASH2, NO) and circulating WBCs (VASH1, VASH2, iNOS) between HNSCC patients and non-cancer controls. This comparison aimed to clarify the physiological and regulatory roles of these molecules in tumor angiogenesis, providing insights into their neovascular behavior and potential for developing novel methods to assess angiogenic activity in cancer patients.

## Materials and methods

### Setting and patient enrollment

This study was approved by the Institutional Review Board of Taipei Veterans General Hospital in Taiwan (IRB-TPEVGH No. 2020–06-012 AC, date of approval: July 10, 2020). It was conducted in accordance with the institutional ethical standards and the Declaration of Helsinki 2013. Written informed consent was obtained from all participants prior to their enrollment.

**Fig. 1:**
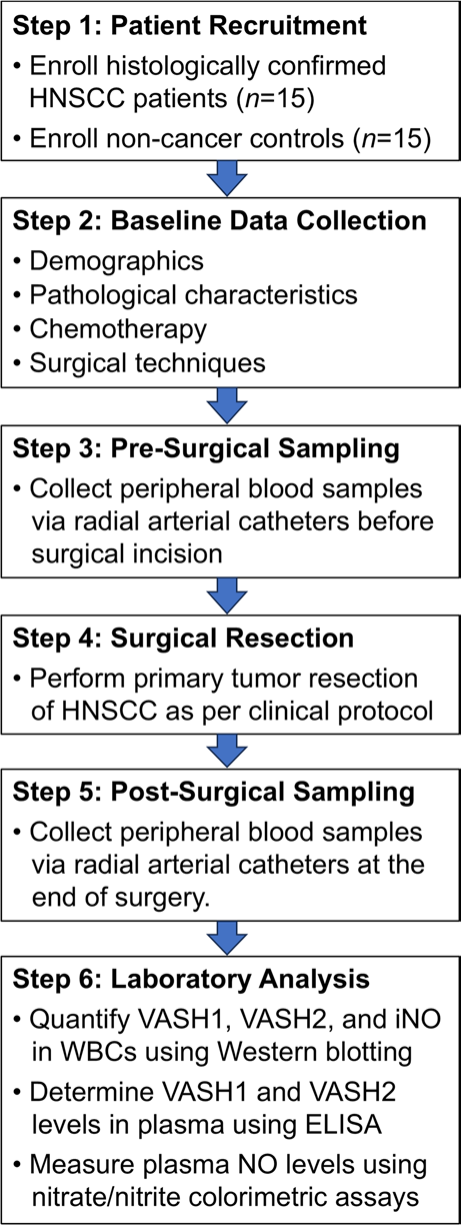
Flowchart illustrating the methodology for studying dynamic changes in vasohibin (VASH) and nitric oxide signaling following surgical resection of head and neck squamous cell carcinoma (HNSCC)

Patients with pathology-confirmed HNSCC who underwent surgical resection of primary tumors at a tertiary medical center between May and November 2021 were recruited for this study. Patients were excluded if they met any of the following criteria: age below 20 years, previous diagnoses of leukocyte disorders (e.g., leukopenia and leukemia), or use of NO therapy (e.g., glyceryl trinitrate) within 30 days before surgery. Following the application of these selection criteria, a total of 15 patients with HNSCC were enrolled. For comparison, 15 age-matched healthy volunteers with no prior cancer diagnoses were recruited as non-cancer controls. This allowed for a comparative analysis of baseline levels of VASH1, VASH2, and NO signaling biomarkers between HNSCC patients and controls. The study methodology is presented in Fig. 1.

### Study outcomes

The primary outcome assessed VASH1 and VASH2 expression levels in peripheral WBCs before and after HNSCC tumor resection. Secondary outcomes included plasma levels of VASH1, VASH2, and NO, and iNOS phosphorylation in WBCs in cancer patients before and after surgery. Baseline levels of these markers were compared between HNSCC patients and non-cancer controls. Using a median VASH1 change fold of 0.72 in leukocytes, we categorized patients into high-deltaVASH1 (≥ 0.72) and low-deltaVASH1 (< 0.72) groups, comparing their 1-year and 3-year overall survival rates, defined as the time from surgery to death from any cause, with data sourced from medical records and death certificates. Survival times for patients without death were censored, and follow-up continued until October 31, 2024.

### Collection of covariates

Clinical data for the cancer patients were obtained from their electronic medical records. Demographic information included age, sex, body mass index, and history of tobacco smoking, alcohol consumption, and betelnut chewing within the 30 days prior to surgery. The American Society of Anesthesiologists physical status classification was used to evaluate their overall health status. Pathological features included primary tumor location (categorized as tongue, buccal mucosa, gingiva, palate, hypopharynx, or other), tumor cell differentiation, angiolymphatic invasion, perineural invasion, and tumor stage [19, 20]. Tumor, node, metastasis staging was categorized into stages I to IV according to the 8 th edition of the American Joint Committee on Cancer (AJCC) criteria [21]. Surgical factors included the use of preoperative chemotherapy, duration of anesthesia, intraoperative blood loss, intraoperative blood transfusion, and surgical procedures (neck dissection and flap reconstruction).

### Blood sample collection

Peripheral blood samples were collected via radial arterial catheters before surgical incision (baseline) and at the end of surgery. Samples were stored in K2 ethylenediaminetetraacetic acid (EDTA)-containing tubes (Becton Dickinson, NJ, USA) for plasma and protein extraction. Plasma was centrifuged at 3000 rpm for 10 min without braking and stored at −80 °C. WBCs were isolated using Ficoll-Paque Plus (GE Healthcare Bio-Sciences AB, Uppsala, Sweden). Equal volumes of WBCs were added to an ice-cold wash buffer containing 1 mM Na_2_HPO_4_, 1 mM EDTA, and 1 mM phenylmethylsulfonyl fluoride (PMSF, Gold Biotechnology, St. Louis, MO, USA), at a pH of 7.4, in a 1:3 ratio. To remove hemoglobin, the WBCs membranes underwent four or more wash cycles, each involving centrifugation at 14,500 rpm for 20 min at 4°C. The washed WBCs membranes were then stored at −80°C for further analysis.

### VASH1 and VASH2 expression

VASH1 and VASH2 proteins were extracted from WBCs, which were lysed using RIPA buffer to ensure efficient lysis and protein solubilization. The protein fraction was isolated via centrifugation. Western blotting was used to analyze the expression levels of VASH1 and VASH2 proteins. Protein samples were separated by SDS-PAGE, transferred to PVDF membranes, and blocked with 5% BSA. The membranes were incubated overnight with primary antibodies specific to VASH1 and VASH2, followed by HRP-conjugated secondary antibodies. An enhanced chemiluminescence (ECL) system was used for detection, and the protein band intensities were quantified using image analysis software to determine the expression levels of VASH1 and VASH2 in the leukocyte samples. To quantify plasma levels of VASH1 and VASH2, enzyme-linked immunosorbent assay (ELISA) kits were employed, following the manufacturer’s protocol, consistent with established methodologies from prior studies [22]. All measurements were performed in duplicate.

### NO levels and iNOS phosphorylation detection

Plasma NO concentration was determined by measuring its stable end products, nitrite and nitrate [23]. Plasma nitrite and nitrate levels were measured using a nitrate/nitrite colorimetric assay kit (Cayman Chemical®, MI, USA). The first step involved converting nitrate to nitrite using nitrate reductase. Subsequently, Griess reagents were added. After 10 min of incubation at room temperature, the absorbance was measured at 550 nm using a microplate reader (BioTek Instruments, VT, USA).

Cytoplasmic proteins were extracted from leukocyte lysates to assess the phosphorylation of iNOS in WBCs. Following lysis of leukocytes with lysis buffer and isolation of the cytoplasmic fraction via centrifugation, iNOS protein was extracted. The phosphorylation levels of iNOS were analyzed using Western blotting. Protein samples were separated by SDS-PAGE, transferred to PVDF membranes, and blocked with 5% BSA. Membranes were incubated overnight with a primary antibody specific to phosphorylated iNOS, followed by an HRP-conjugated secondary antibody. An ECL system was used for detection, and the intensity of the protein bands was quantified using image analysis software to determine iNOS phosphorylation levels in the leukocyte samples. All measurements were performed in duplicate to determine the mean value.

### Statistical analysis

A minimum sample size of 14 was determined to achieve 80% power to detect an 80% change in VASH1 or VASH2 levels in WBCs for paired comparisons, using a two-sided significance level of 0.05. Kolmogorov–Smirnov tests assessed normality. Independent t-tests or Mann–Whitney U tests compared baseline plasma levels of VASH1, VASH2, NO, and iNOS phosphorylation in WBCs between cancer patients and non-cancer controls, as appropriate. Paired t-tests or Wilcoxon signed-rank tests evaluated changes in these markers before and after surgery in HNSCC patients, as appropriate. All hypothesis tests used a two-sided significance level of 0.05. Statistical analyses were conducted using SAS version 9.4 (SAS Institute Inc., Cary, NC, USA), and graphs were created with Prism version 5.0 (GraphPad Software, San Diego, CA, USA).

## Results

### Baseline patient characteristics

Table 1 presents the clinical characteristics of the included cancer patients. The average age of the cancer patients was 54.7 ± 11.2 years, with 12 (80.0%) being male. Regarding pathology, the primary tumor was located in the buccal mucosa in 5 (33.3%) patients, and 6 (40.0%) patients had stage IV cancer. Fourteen patients (93.3%) had moderately-differentiated tumors, and 5 (33.3%) and 6 (40.0%) patients had angiolymphatic invasion and perineural invasion, respectively. Nine patients (60.0%) underwent neck dissection to remove lymph nodes from the neck, and 6 (40.0%) underwent free flap reconstruction. In the non-cancer control group, the mean age was 58.7 ± 9.7 years, with 13 (86.7%) participants being male.

**Table 1:** Clinical characteristics of the included cancer patients

	**Cancer patients** **(*n* = 15)**
**Age, year**	54.7 ± 11.2
**Sex, male**	12 (80.0%)
**Body weight, kg**	74.8 ± 14.2
**Body height, cm**	169.3 ± 5.8
**Body mass index, kg·m**^**−2**^	26.1 ± 5.1
**ASA class**	
II	8 (53.3%)
III	7 (46.7%)
**Tobacco smoking**	7 (46.7%)
**Alcohol consumption**	6 (40.0%)
**Betelnut chewing**	8 (53.3%)
**Primary tumor location**	
Tongue	3 (20.0%)
Buccal mucosa	5 (33.3%)
Gingiva	2 (13.3%)
Palate	1 (6.7%)
Hypopharynx	1 (6.7%)
Other	3 (20.0%)
**TNM classification**	
T1	4 (26.7%)
T2	2 (13.3%)
T3	4 (26.7%)
T4a	2 (13.3%)
T4b	3 (20.0%)
N0	11 (73.3%)
N1	1 (6.7%)
N2b	1 (6.7%)
N2c	1 (6.7%)
N3b	1 (6.7%)
M0	15 (100.0%)
**Cancer stage**	
I	4 (26.7%)
II	2 (13.3%)
III	3 (20.0%)
IVa	3 (20.0%)
IVb	3 (20.0%)
**Differentiation grade**	
Good	1 (6.7%)
Moderate	14 (93.3%)
Poor	0 (0)
**Angiolymphatic invasion**	5 (33.3%)
**Perineural invasion**	6 (40.0%)
**Preoperative chemotherapy**	1 (6.7%)
**Neck dissection**	9 (60.0%)
**Flap reconstruction**	6 (40.0%)

Values were mean ± standard deviation or counts (percent)

*ASA* American Society of Anesthesiologists, *TNM* tumor, node, metastasis

Table 2 presents intraoperative vital signs and laboratory testing data. The duration of anesthesia was 680 ± 235 min. The intraoperative blood loss volume was 600 ± 261 mL, and 3 (20.0%) and 1 (6.6%) patient received red blood cell and fresh frozen plasma transfusions during surgery, respectively.

**Table 2:** Intraoperative vital signs and laboratory testing data of the cancer patients

	**Pre-induction**	**End of surgery**
**Systolic blood pressure, mmHg**	122 ± 15	138 ± 16
**Diastolic blood pressure, mmHg**	69 ± 15	69 ± 15
**Heart rate, beats·min**^**−1**^	81 ± 10	77 ± 17
**Body temperature, ℃**	36.0 ± 0.2	36.2 ± 0.3
**SpO**_**2**_**, %**	99 ± 1	99 ± 1
**Serum glucose, mg·dL**^**−1**^	164 ± 53	165 ± 38
**Serum hemoglobin, g·dL**^**−1**^	12.6 ± 1.5	13.5 ± 0.9
	**Intraoperative parameters**	
**Anesthesia duration, min**	680 ± 235	
**Surgical blood loss, mL**	600 ± 261	
**Blood transfusion**		
Red blood cells	3 (20.0%)	
Fresh frozen plasma	1 (6.6%)	

Values were mean ± standard deviation or counts (percent). SpO_2:_ oxyhemoglobin saturation by pulse oximetry

### Baseline levels of VASH1 and VASH2

**Fig. 2:**
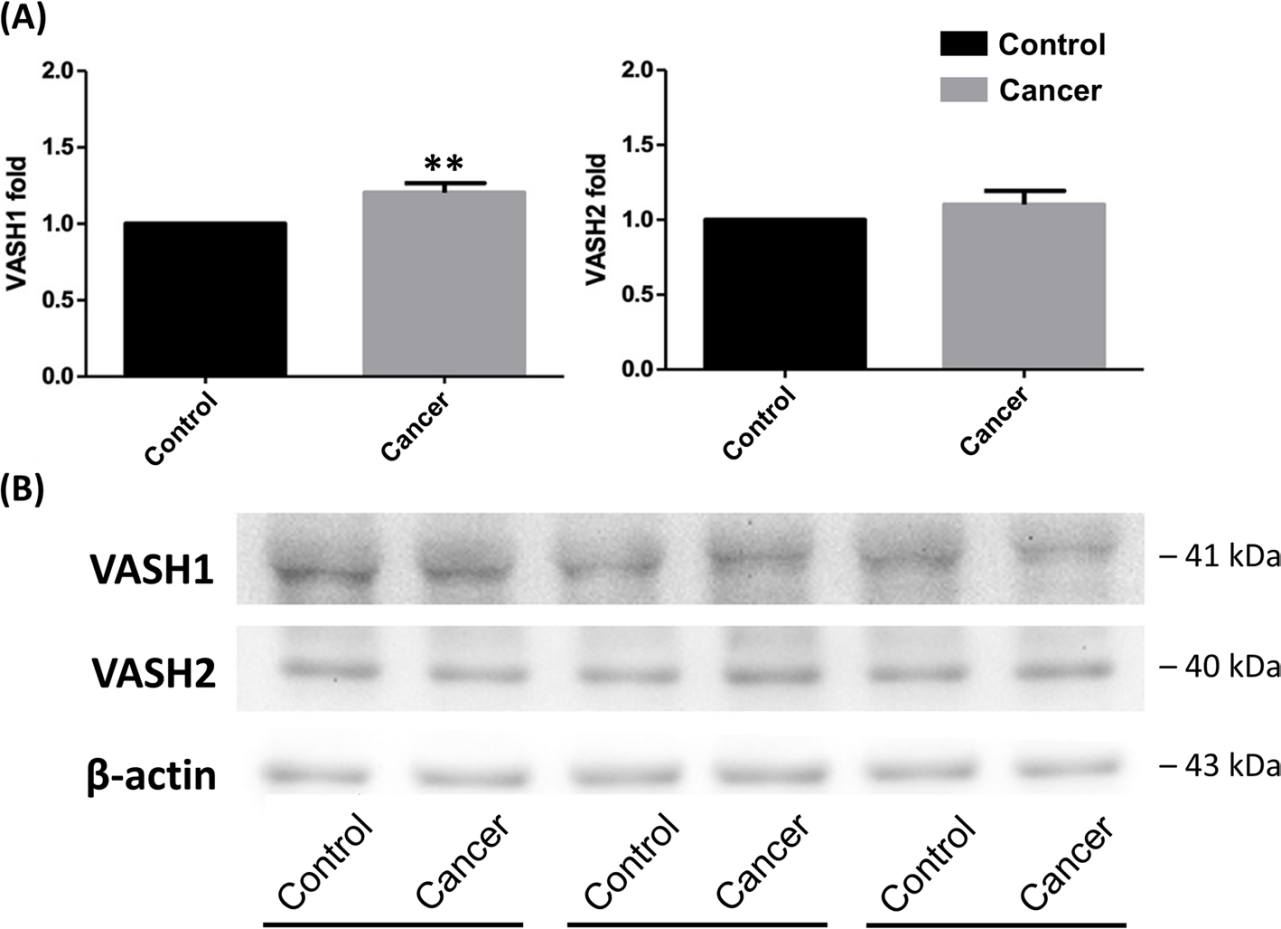
**A** Baseline vasohibin-1 levels in WBCs were significantly higher in cancer patients compared to controls. However, there was no significant difference in vasohibin-2 levels in WBCs between the two groups. Data were presented as mean ± SD. (^**^P < 0.01 indicate a significant difference between cancer and non-cancer subjects). **B** A representative gel was shown. VASH1 = vasohibin-1; VASH2 = vasohibin-2

Plasma VASH1 concentration was significantly higher in cancer patients compared to non-cancer controls (2425 ± 1493 pg·mL^−1^ vs. 1402 ± 368 pg·mL^−1^, *p* = 0.0097 by Mann–Whitney U tests; Kolmogorov–Smirnov test, *p* < 0.0100). However, no significant difference was observed in plasma VASH2 levels between the two groups. The expression level of VASH1 in WBCs was significantly higher in cancer patients compared to non-cancer controls (1.24 ± 0.06 folds, *p* = 0.0093 by independent t-tests; Kolmogorov–Smirnov test, *p* > 0.1500) (Fig. 2 and supplementary Figure S1). However, no significant difference was observed in VASH2 expression levels in WBCs between cancer patients and controls (1.15 ± 0.37 folds, *p* = 0.3030 by Mann–Whitney U tests; Kolmogorov–Smirnov test, *p* < 0.0100).

### Baseline plasma NO level and WBCs iNOS phosphorylation

**Fig. 3:**
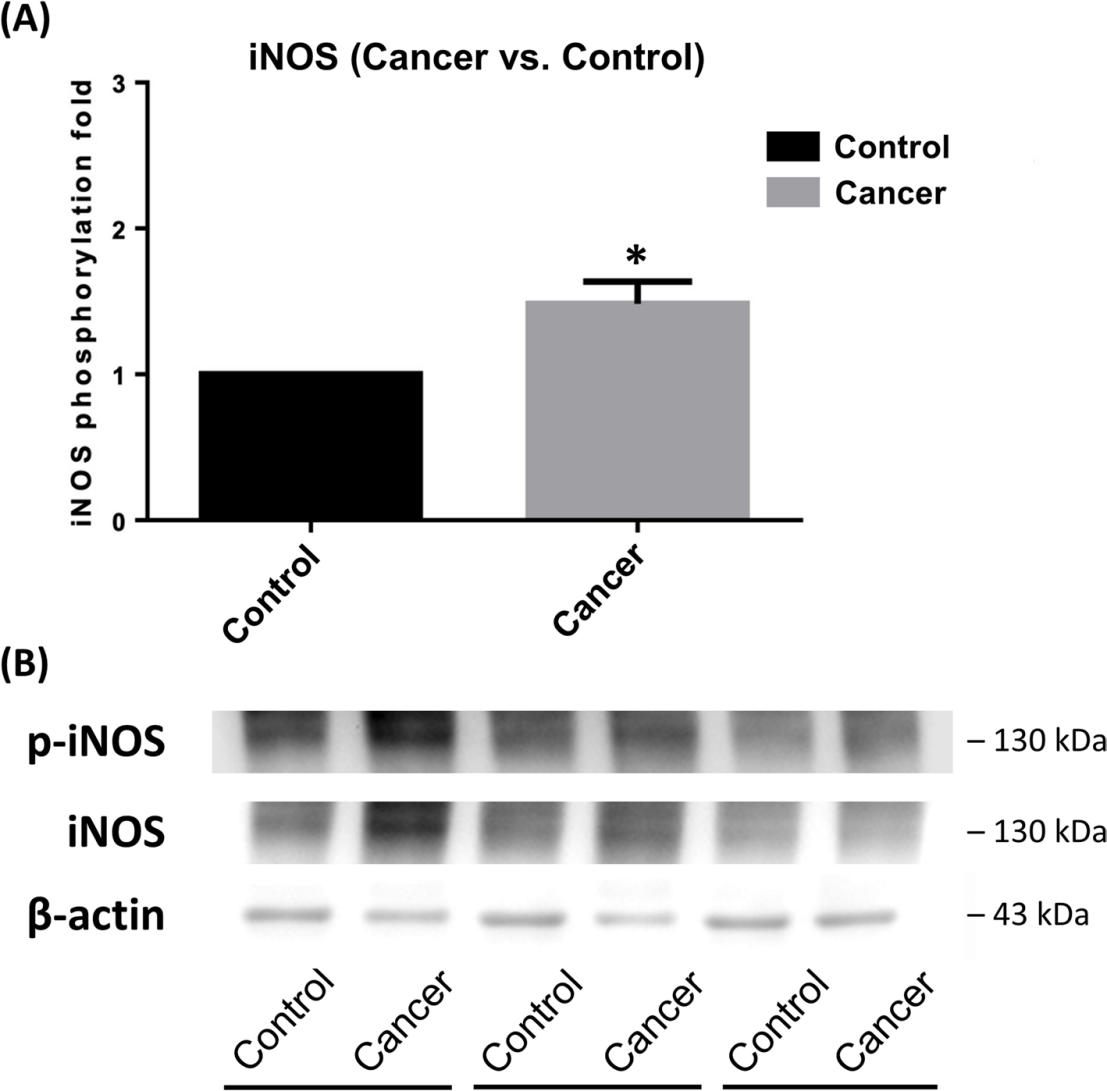
**A** The baseline level of iNOS phosphorylation in WBCs was significantly higher in cancer patients compared to controls. Data were presented as mean ± SD. (^*^P < 0.05 indicate a significant difference between cancer and non-cancer subjects). **B** A representative gel was shown

Plasma NO levels were significantly elevated in cancer patients compared to non-cancer controls, with means of 6.04 ± 2.23 μM versus 2.21 ± 1.62 μM (*p* = 0.0004 by Mann–Whitney U tests; Kolmogorov–Smirnov test, *p* < 0.0100), respectively. Additionally, the degree of phosphorylation expression of leukocyte-induced iNOS was significantly higher in cancer patients compared to non-cancer controls (1.48 ± 0.12 folds, *p* = 0.0235 by independent t-tests; Kolmogorov–Smirnov test, *p* > 0.1500) (Fig. 3 and supplementary Figure S2).

### Dynamic changes in VASH1 and VASH2

**Fig. 4:**
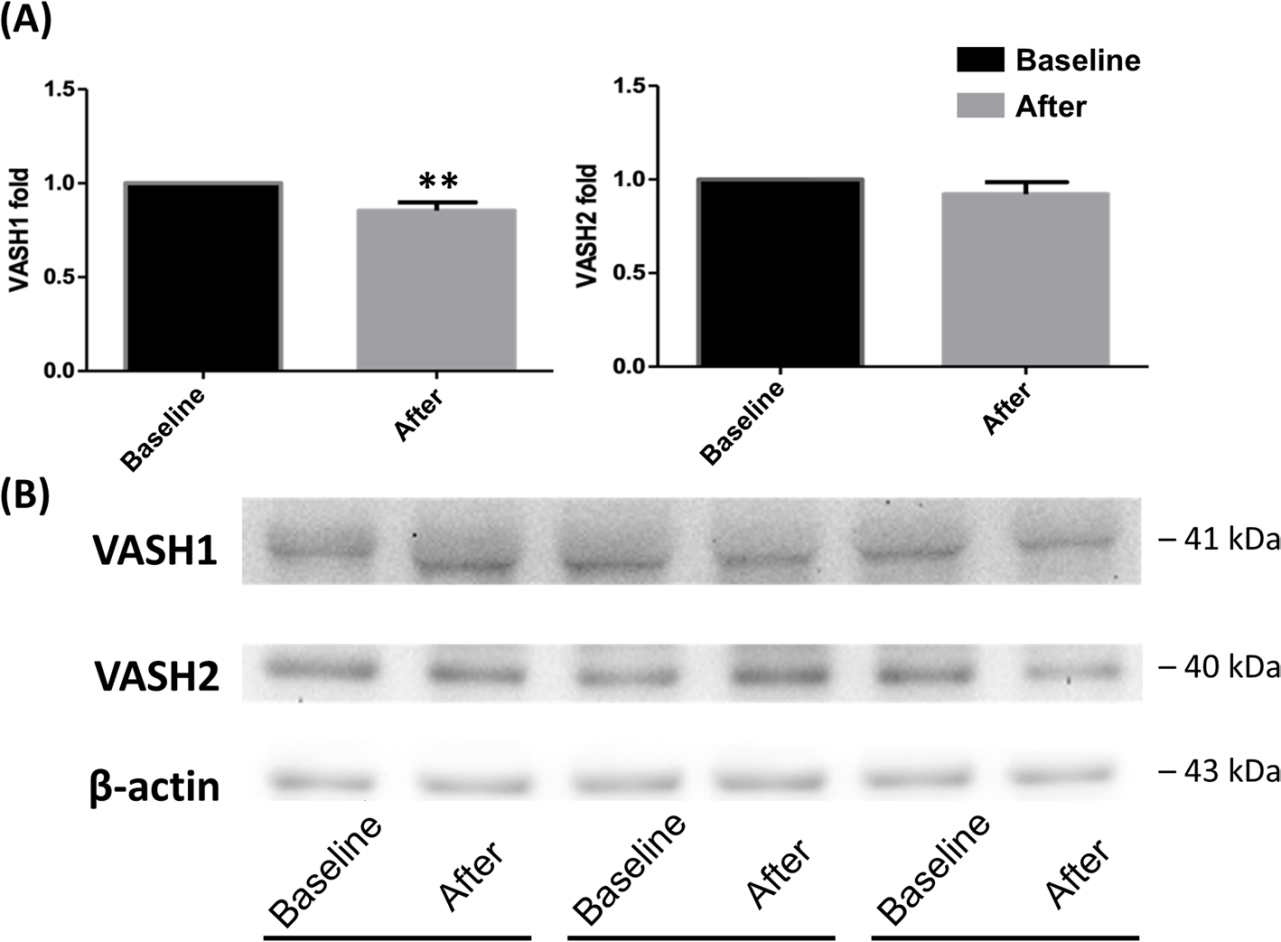
**A** Vasohibin-1 expression in WBCs was significantly reduced after tumor resection in cancer patients. However, no significant change was observed in vasohibin-2 expression levels. Data were presented as mean ± SD. (^**^P < 0.01 indicate a significant difference before and after surgery). **B** A representative gel was shown. VASH1 = vasohibin-1; VASH2 = vasohibin-2

Plasma VASH1 levels were significantly reduced after tumor resection, 2233 ± 1464 pg·m^−1^ vs. 2425 ± 1493 pg·m^−1^ (0.90 ± 0.07 fold, *p* = 0.0085 by Wilcoxon signed-rank test; Kolmogorov–Smirnov test, *p* < 0.0100) (supplementary Figure S3). However, no significant change in plasma VASH2 levels was observed before and after surgery (0.94 ± 0.13 fold, *p* = 0.0980 by independent t-tests; Kolmogorov–Smirnov test, *p* > 0.1500). VASH1 expression levels in WBCs were significantly reduced after tumor resection (0.85 ± 0.04 fold, *p* = 0.0068 by Wilcoxon signed-rank test; Kolmogorov–Smirnov test, *p* < 0.0100) (Fig. 4 and supplementary Figure S4), while VASH2 expression levels did not change significantly after surgery (0.92 ± 0.11 fold, *p* = 0.4780 by Wilcoxon signed-rank test; Kolmogorov–Smirnov test, *p* < 0.0100). The 1-year and 3-year overall survival rates were 100.0% (95% CI: 100.0–100.0) and 100.0% (95% CI: 100.0–100.0) in the high-deltaVASH1 group, while the low-deltaVASH1 group had rates of 100.0% (95% CI: 100.0–100.0; *p* > 0.9999 by log-rank test) and 85.7% (95% CI: 63.3–100.0; *p* = 0.4795), respectively.

### Dynamic changes in plasma NO and WBCs iNOS phosphorylation

**Fig. 5:**
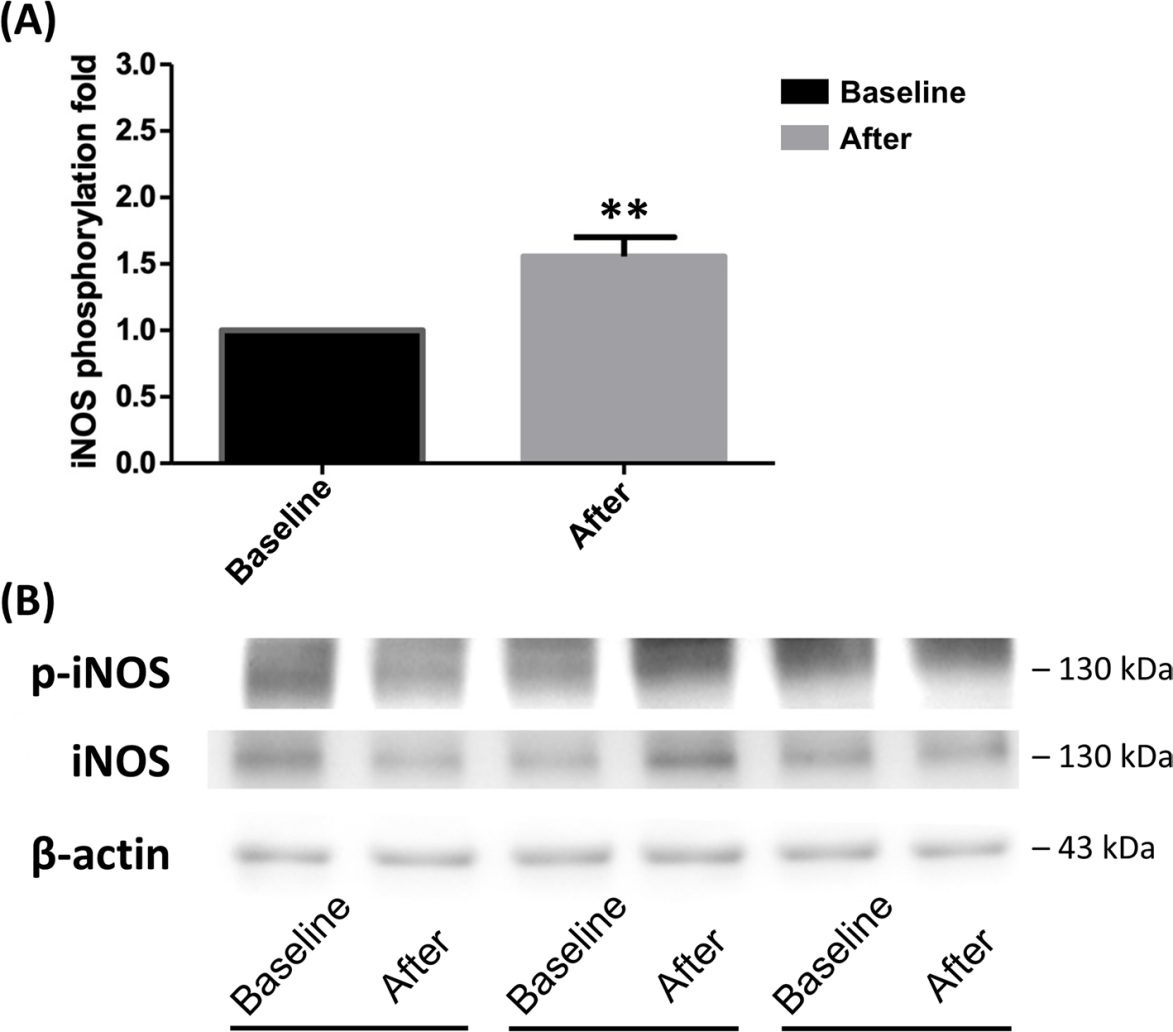
**A** The phosphorylation level of iNOS in WBCs was significantly enhanced after tumor resection in the cancer patients. Data were presented as mean ± SD. (^**^P < 0.01 indicate a significant difference before and after surgery). **B** A representative gel was shown

Plasma NO concentration was significantly reduced in cancer patients after surgery (0.29 ± 0.09 fold, *p* = 0.0001 by Wilcoxon signed-rank test; Kolmogorov–Smirnov test, *p* < 0.0100). Additionally, the degree of phosphorylation expression of iNOS in WBCs increased significantly after surgery (1.52 ± 0.10 fold, *p* = 0.0024 by Wilcoxon signed-rank test; Kolmogorov–Smirnov test, *p* < 0.0100) (Fig. 5 and supplementary Figure S5).

## Discussion

This study revealed that VASH1 levels in WBCs and plasma were significantly elevated in patients with HNSCC, but the baseline level of VASH2 was similar between HNSCC patients and controls. Following surgical resection of HNSCC tumors, plasma and leukocyte levels of VASH1, but not VASH2, were significantly reduced, accompanied by a decrease in plasma NO. These findings may serve for monitoring the efficacy of therapeutic interventions for malignant tumors, particularly HNSCC.

Our study revealed significantly higher VASH1 levels in HNSCC patients compared to non-cancer controls, with no notable difference in VASH2 levels, reflecting their distinct roles in the tumor microenvironment. VASH1, primarily expressed in endothelial cells, is upregulated by pro-angiogenic factors like VEGF, which are often overexpressed in tumors to drive angiogenesis, likely acting as a feedback mechanism to regulate excessive vessel formation [2–4]. In contrast, VASH2, expressed in cancer cells or infiltrating mononuclear cells, may not be consistently elevated due to tumor heterogeneity or variable immune cell infiltration [5–8]. Additionally, post-surgical comparisons showed a significant decrease in VASH1 levels in WBCs and plasma, while VASH2 levels remained unchanged. This likely stems from the removal of the tumor-driven angiogenic microenvironment, where tumors secrete factors like VEGF that stimulate VASH1 and iNOS expression in WBCs, enhancing NO production to support vascularization [2–4]. Surgical resection removes this stimulus, diminishing inflammatory and angiogenic signals that activate these pathways.

The vasohibin family significantly influences tumor angiogenesis regulation [24]. Kopczynska et al. demonstrated that surgical resection of non-small cell lung cancer altered angiogenic factor levels, with angiotensin II and VEGF rising by postoperative day 7 and declining by day 30 [25]. However, prior studies have not explored whether tumor resection specifically affects VASH1 or VASH2 concentrations in cancer patients. VASH1 has emerged as a potential prognostic biomarker for HNSCC, with Torii et al. linking its expression in tumor cells to increased lymph node recurrence [12]. In prostate cancer, VASH1 density independently predicts prostate-specific antigen recurrence [26], and its expression correlates with tumor progression, metastasis, and micro-vessel density across cancers [27, 28]. Recombinant adenovirus encoding vasohibin shows promise in suppressing tumor angiogenesis and growth [29]. Although VASH1 holds promise, the search for predictive biomarkers in HNSCC persists, particularly focusing on immune checkpoint inhibitors targeting the programmed cell death protein-1 pathway [30]. Our research suggests vasohibin activation in WBCs could serve as a novel biomarker for assessing angiogenic activity, tracking metastasis, and evaluating chemotherapy efficacy, enhancing precision in HNSCC diagnosis and treatment alongside established markers [31–33]. Further studies are needed to validate the prognostic value of vasohibin and identify additional biomarkers for targeted therapies and immunotherapies.

The lack of significant changes in VASH2 levels following surgical resection of HNSCC may arise from multiple factors. In contrast to VASH1, which is closely linked to tumor progression and angiogenesis, VASH2 may play a more restricted or context-dependent role within the HNSCC tumor microenvironment. For instance, VASH2 expression might be primarily governed by local microenvironmental signals, such as hypoxia or cytokine profiles, which remain largely unaffected by resection [34, 5]. Alternatively, VASH2 may predominantly influence early tumor development, with its expression stabilizing in advanced HNSCC, resulting in minimal responsiveness to surgical intervention [35]. This stability suggests that VASH2 may not be a primary driver of angiogenesis regulation in this context, potentially diminishing its value as a therapeutic target or biomarker in advanced HNSCC. Further research is essential to elucidate the specific role of VASH2 and its interactions with other angiogenic factors, providing deeper insights into its implications for tumor behavior and vascular remodeling in HNSCC.

In our study, HNSCC patients displayed significantly higher plasma NO metabolite levels than non-cancer controls, with a notable reduction after tumor resection. This reduction possibly correlates with reduced VASH1 levels, driven primarily by endothelial NO synthase (eNOS) phosphorylation rather than iNOS activity measured in WBCs [36]. Intriguingly, iNOS phosphorylation in WBCs increased after surgery, contrasting with the decline in plasma NO metabolites, suggesting attenuated vasohibin and NO signaling in WBCs and a normalization of plasma NO levels toward those of non-cancer individuals. NO signaling influences HNSCC angiogenesis and tumor behavior in complex ways. At low levels, eNOS-derived NO from tumor or endothelial cells promotes angiogenesis by enhancing VEGF expression and endothelial cell proliferation, supporting tumor growth [37]. Conversely, high iNOS-derived NO levels from immune cells, like macrophages, may exert cytotoxic effects, inducing tumor cell apoptosis and inhibiting progression [37]. Additionally, NO modulates the tumor microenvironment by regulating immune cell infiltration and cytokine production, potentially fostering immunosuppression or enhancing anti-tumor responses [38]. The interplay between NO levels, cellular sources, and the HNSCC microenvironment underscores its dual role, necessitating further research to elucidate mechanisms and therapeutic potential.

Assessing angiogenesis-regulating markers, including VASH1, VASH2, and iNOS, in circulating WBCs provides deeper insights into the tumor microenvironment and immune response in HNSCC, surpassing the limitations of plasma measurements alone. Prior research indicates that WBCs and platelets in cancer patients harbor significantly higher VEGF levels than in healthy controls, contributing to tumor angiogenesis and metastasis [39]. While angiogenic factors like basic fibroblast growth factor and angiogenin show potential, their clinical utility is less established than VEGF [40]. In acute myeloid leukemia, elevated cellular VEGF levels independently predict shorter survival [41]. In contrast to plasma, which measures systemic levels of VASH1, VASH2, and NO, circulating WBCs actively contribute to tumor-associated immune and angiogenic activities, expressing these markers in direct response to tumor signals. This dynamic expression offers a direct view of cellular mechanisms driving tumor progression, capturing localized activity that plasma may obscure. Our study found that VASH1 and NO signaling in WBCs decreased after HNSCC tumor resection, highlighting their potential for monitoring therapeutic responses.

This study has several limitations. First, we did not monitor long-term postoperative changes in VASH1 and VASH2 levels. Second, we did not assess the relationship between vasohibin levels and cancer recurrence risk, leaving uncertainty about their predictive value for oncological outcomes in HNSCC. Third, the modest sample size may have limited statistical power for certain outcomes and restricted comprehensive multivariable adjustments. Fourth, the mechanisms underlying how dynamic changes in vasohibin and NO levels in WBCs affect angiogenesis or tumor behavior remain unclear, necessitating further in vitro or in vivo studies to explore interactions with the tumor microenvironment following surgery or anticancer therapy. Finally, the observational study design may introduce confounding from unmeasured or unadjusted factors, such as immune-modifying drugs or anesthesia management.

The findings of this study offer several perspectives for guiding future research in HNSCC and tumor angiogenesis. First, the observed dynamic changes in VASH1, VASH2, and NO signaling in WBCs and plasma following surgical resection highlight their potential as biomarkers for monitoring angiogenic activity and therapeutic responses. These insights pave the way for longitudinal studies to track vasohibin and NO levels over extended postoperative periods to assess their predictive value for oncological outcomes. Second, the distinct roles of VASH1 and VASH2 in the tumor microenvironment suggest avenues for investigating their interactions with other angiogenic factors, such as VEGF, and immune checkpoint pathways to optimize targeted therapies and immunotherapies. Last, future research should also focus on larger, controlled studies to validate these biomarkers, elucidate underlying molecular mechanisms, and explore their utility in personalized treatment strategies, ultimately enhancing precision medicine for HNSCC patients.

## Conclusions

This study identified angiogenesis regulators as potential biomarkers in HNSCC patients. We observed downregulation of VASH1 and NO in plasma and VASH1 in circulating WBCs following curative surgical resection, while iNOS levels were upregulated in WBCs. Compared to non-cancer controls, HNSCC patients showed elevated VASH1 and NO in plasma and VASH1 and iNOS in WBCs. These findings suggest that these biomarkers may hold prognostic and predictive value in HNSCC. However, further studies are required to validate these results and assess their clinical utility in oncology practice.

## Supplementary Information

Supplementary Material 1.

## Data Availability

No datasets were generated or analysed during the current study.

## Abbreviations

AJCC: American Joint Committee on Cancer
BSA: Bovine serum albumin
ECL: Enhanced chemiluminescence
ECs: Endothelial cells
EDTA: Ethylenediaminetetraacetic acid
HNSCC: Head and neck squamous cell carcinoma
iNOS: Inducible nitric oxide synthase
IRB: Institutional review board
NO: Nitric oxide
eNOS: Endothelial nitric oxide synthase
PMSF: Phenylmethylsulfonyl fluoride
PVDF: Polyvinylidene difluoride
SpO_2_: Oxyhemoglobin saturation
VASH1: Vasohibin-1
VASH2: Vasohibin-2
VEGF: Vascular endothelial growth factor
VEGFR: Vascular endothelial growth factor receptor
WBCs: White blood cells

## References

[1] De Palma M, Biziato D, Petrova TV. Microenvironmental regulation of tumour angiogenesis. Nat Rev Cancer. 2017;17:457–74. 28706266 10.1038/nrc.2017.51

[2] Watanabe K, Hasegawa Y, Yamashita H, Shimizu K, Ding Y, Abe M, et al. Vasohibin as an endothelium-derived negative feedback regulator of angiogenesis. J Clin Invest. 2004;114:898–907. 15467828 10.1172/JCI21152 PMC518662

[3] Zhao G, Na R, Li L, Xiao H, Ding N, Sun Y, et al. Vasohibin-1 inhibits angiogenesis and suppresses tumor growth in renal cell carcinoma. Oncol Rep. 2017;38:1021–8. 28656230 10.3892/or.2017.5746

[4] Hosaka T, Kimura H, Heishi T, Suzuki Y, Miyashita H, Ohta H, et al. Vasohibin-1 expression in endothelium of tumor blood vessels regulates angiogenesis. Am J Pathol. 2009;175:430–9. 19498005 10.2353/ajpath.2009.080788 PMC2708828

[5] Iida-Norita R, Kawamura M, Suzuki Y, Hamada S, Masamune A, Furukawa T, et al. Vasohibin-2 plays an essential role in metastasis of pancreatic ductal adenocarcinoma. Cancer Sci. 2019;110:2296–308. 31074083 10.1111/cas.14041 PMC6609860

[6] Kitahara S, Suzuki Y, Morishima M, Yoshii A, Kikuta S, Shimizu K, et al. Vasohibin-2 modulates tumor onset in the gastrointestinal tract by normalizing tumor angiogenesis. Mol Cancer. 2014;13:99. 24885408 10.1186/1476-4598-13-99 PMC4113181

[7] Xue X, Zhang Y, Zhi Q, Tu M, Xu Y, Sun J, et al. MiR200-upregulated Vasohibin 2 promotes the malignant transformation of tumors by inducing epithelial-mesenchymal transition in hepatocellular carcinoma. Cell Commun Signal. 2014;12:62. 25269476 10.1186/s12964-014-0062-x PMC4195883

[8] Takahashi Y, Koyanagi T, Suzuki Y, Saga Y, Kanomata N, Moriya T, et al. Vasohibin-2 expressed in human serous ovarian adenocarcinoma accelerates tumor growth by promoting angiogenesis. Mol Cancer Res. 2012;10:1135–46. 22826464 10.1158/1541-7786.MCR-12-0098-T

[9] Girotti AW, Fahey JF, Korytowski W. Role of nitric oxide in hyper-aggressiveness of tumor cells that survive various anti-cancer therapies. Crit Rev Oncol Hematol. 2022;179:103805. 36087851 10.1016/j.critrevonc.2022.103805

[10] Dios-Barbeito S, González R, Cadenas M, García LF, Victor VM, Padillo FJ, et al. Impact of nitric oxide in liver cancer microenvironment. Nitric Oxide. 2022;128:1–11. 35940533 10.1016/j.niox.2022.07.006

[11] Shen Z, Kauttu T, Seppänen H, Vainionpää S, Ye Y, Wang S, et al. Vasohibin-1 and vasohibin-2 expression in gastric cancer cells and TAMs. Med Oncol. 2012;29:2718–26. 22438034 10.1007/s12032-012-0212-1

[12] Torii C, Hida Y, Shindoh M, Akiyama K, Ohga N, Maishi N, et al. Vasohibin-1 as a novel prognostic factor for head and neck squamous cell carcinoma. Anticancer Res. 2017;37:1219–25. 28314285 10.21873/anticanres.11437

[13] Sano R, Kanomata N, Suzuki S, Shimoya K, Sato Y, Moriya T, et al. Vasohibin-1 is a poor prognostic factor of ovarian carcinoma. Tohoku J Exp Med. 2017;243:107–14. 29057763 10.1620/tjem.243.107

[14] Kanomata N, Sato Y, Miyaji Y, Nagai A, Moriya T. Vasohibin-1 is a new predictor of disease-free survival in operated patients with renal cell carcinoma. J Clin Pathol. 2013;66:613–9. 23543668 10.1136/jclinpath-2013-201444

[15] Wang Q, Tian X, Zhang C, Wang Q. Upregulation of vasohibin-1 expression with angiogenesis and poor prognosis of hepatocellular carcinoma after curative surgery. Med Oncol. 2012;29:2727–36. 22101788 10.1007/s12032-011-0106-7

[16] Tamaki K, Sasano H, Maruo Y, Takahashi Y, Miyashita M, Moriya T, et al. Vasohibin-1 as a potential predictor of aggressive behavior of ductal carcinoma in situ of the breast. Cancer Sci. 2010;101:1051–8. 20704578 10.1111/j.1349-7006.2009.01483.x PMC11158447

[17] Vakkala M, Kahlos K, Lakari E, Pääkkö P, Kinnula V, Soini Y. Inducible nitric oxide synthase expression, apoptosis, and angiogenesis in in situ and invasive breast carcinomas. Clin Cancer Res. 2000;6:2408–16. 10873093

[18] Wu HL, Chu YH, Tai YH, Tsou MY, Wu CH, Lo WL, et al. Stage-dependent angiopoietin-Tie2 and nitric oxide signaling of erythrocytes in response to surgical trauma in head and neck cancer. World J Surg Oncol. 2020;18:209. 32799882 10.1186/s12957-020-01991-9 PMC7429775

[19] Jones HB, Sykes A, Bayman N, Sloan P, Swindell R, Patel M, et al. The impact of lymphovascular invasion on survival in oral carcinoma. Oral Oncol. 2009;45:10–5. 18620889 10.1016/j.oraloncology.2008.03.009

[20] Schmitd LB, Scanlon CS, D’Silva NJ. Perineural invasion in head and neck cancer. J Dent Res. 2018;97:742–50. 29443582 10.1177/0022034518756297 PMC6728584

[21] Zanoni DK, Patel SG, Shah JP. Changes in the 8th Edition of the American Joint Committee on Cancer (AJCC) Staging of Head and Neck Cancer: Rationale and Implications. Curr Oncol Rep. 2019;21:52. 30997577 10.1007/s11912-019-0799-x PMC6528815

[22] Suzuki Y, Ito O, Kohzuki M, Ichiki M, Persistent Physical YS. Exercise rises the plasma concentration of vasohibin-1 in patients with peripheral vascular disease. Gen Intern Med Clin Innov. 2016;1:1–4.

[23] Tai YH, Chu YH, Wu HL, Lin SM, Tsou MY, Huang CH, et al. High-dose nitroglycerin administered during rewarming preserves erythrocyte deformability in cardiac surgery with cardiopulmonary bypass. Microcirculation. 2020;27:e12608. 31991513 10.1111/micc.12608

[24] Du H, Zhao J, Hai L, Wu J, Yi H, Shi Y. The roles of vasohibin and its family members: beyond angiogenesis modulators. Cancer Biol Ther. 2017;18:827–32. 28886304 10.1080/15384047.2017.1373217 PMC5710674

[25] Kopczyńska E, Dancewicz M, Kowalewski J, Makarewicz R, Kardymowicz H, Kaczmarczyk A, et al. Time-dependent changes of plasma concentrations of angiopoietins, vascular endothelial growth factor, and soluble forms of their receptors in nonsmall cell lung cancer patients following surgical resection. ISRN Oncol. 2012;2012:638352. 22550599 10.5402/2012/638352 PMC3324894

[26] Kosaka T, Miyazaki Y, Miyajima A, Mikami S, Hayashi Y, Tanaka N, et al. The prognostic significance of vasohibin-1 expression in patients with prostate cancer. Br J Cancer. 2013;108:2123–9. 23591203 10.1038/bjc.2013.169 PMC3670477

[27] Zhang B, Wu Z, Xie W, Tian D, Chen F, Qin C, et al. The expression of vasohibin-1 and its prognostic significance in bladder cancer. Exp Ther Med. 2017;14:3477–84. 29042936 10.3892/etm.2017.4969 PMC5639433

[28] Liu S, Han B, Sun M, Wang J, Sun Y, Wang Y. Significance of vasohibin 1 in cancer patients: a systematic review and meta analysis. J Cancer Res Ther. 2022;18:567–75. 35645129 10.4103/jcrt.jcrt_281_21

[29] Li D, Zhou K, Wang S, Shi Z, Yang Z. Recombinant adenovirus encoding vasohibin prevents tumor angiogenesis and inhibits tumor growth. Cancer Sci. 2010;101:448–52. 19886910 10.1111/j.1349-7006.2009.01388.x PMC11158149

[30] Elbehi AM, Anu RI, Ekine-Afolabi B, Cash E. Emerging role of immune checkpoint inhibitors and predictive biomarkers in head and neck cancers. Oral Oncol. 2020;109:104977. 32853912 10.1016/j.oraloncology.2020.104977

[31] Xu Z, Zhang M, Guo Z, Chen L, Yang X, Li X, et al. Stemness-related lncRNAs signature as a biologic prognostic model for head and neck squamous cell carcinoma. Apoptosis. 2023;28:860–80. 36997733 10.1007/s10495-023-01832-6

[32] Zhang M, Sun Q, Han Z, Qin X, Gao T, Xu Y, et al. Construction of a novel disulfidptosis-related lncRNAs signature for prognosis prediction and anti-tumor immunity in laryngeal squamous cell carcinoma. Heliyon. 2024;10:e30877. 38774325 10.1016/j.heliyon.2024.e30877 PMC11107247

[33] Liu J, Sun Q, Zhao J, Qin X, Gao T, Bai G, Chen G, Guo Z. Early death in supraglottic laryngeal squamous cell carcinoma: a population-based study. Ear Nose Throat J. 2024;103(10):650–8. 35171058 10.1177/01455613221078184

[34] Tu M, Li Z, Liu X, Lv N, Xi C, Lu Z, et al. Vasohibin 2 promotes epithelial-mesenchymal transition in human breast cancer via activation of transforming growth factor β 1 and hypoxia dependent repression of GATA-binding factor 3. Cancer Lett. 2017;388:187–97. 27867016 10.1016/j.canlet.2016.11.016

[35] Hara H, Ozawa S, Ninomiya Y, Yamamoto M, Ogimi M, Nabeshima K, et al. Prognostic significance of vasohibin-1 and vasohibin-2 immunohistochemical expression in gastric cancer. Surg Today. 2020;50:1530–43. 32494966 10.1007/s00595-020-02040-4

[36] Xu W, Liu LZ, Loizidou M, Ahmed M, Charles IG. The role of nitric oxide in cancer. Cell Res. 2002;12:311–20. 12528889 10.1038/sj.cr.7290133

[37] Holotiuk VV, Kryzhanivska AY, Churpiy IK, Tataryn BB, Ivasiutyn DY. Role of nitric oxide in pathogenesis of tumor growth and its possible application in cancer treatment. Exp Oncol. 2019;41:210–5. 31569933 10.32471/exp-oncology.2312-8852.vol-41-no-3.13515

[38] Khan FH, Dervan E, Bhattacharyya DD, McAuliffe JD, Miranda KM, Glynn SA. The role of nitric oxide in cancer: master regulator or NOt? Int J Mol Sci. 2020;21:9393. 33321789 10.3390/ijms21249393 PMC7763974

[39] Poon RT, Fan ST, Wong J. Clinical implications of circulating angiogenic factors in cancer patients. J Clin Oncol. 2001;19:1207–25. 11181687 10.1200/JCO.2001.19.4.1207

[40] Aguayo A, Estey E, Kantarjian H, Mansouri T, Gidel C, Keating M, et al. Cellular vascular endothelial growth factor is a predictor of outcome in patients with acute myeloid leukemia. Blood. 1999;94:3717–21. 10572084

[41] Salven P, Orpana A, Joensuu H. Leukocytes and platelets of patients with cancer contain high levels of vascular endothelial growth factor. Clin Cancer Res. 1999;5:487–91. 10100697

