# Supplementary material for: Dynamic changes in vasohibin and nitric oxide signaling following surgical resection of head and neck squamous cell carcinoma
Source: World J Surg Oncol. 2025 Jun 7;23:221. doi: 10.1186/s12957-025-03853-8 (PMC12144735; doi:10.1186/s12957-025-03853-8)

**Figure S1.** The full uncropped image of representative gels of baseline vasohibin-1 (A), vasohibin-2 (B) in WBCs, β-actin (C), and actin maker (D)

**(A) Vasohibin-1**


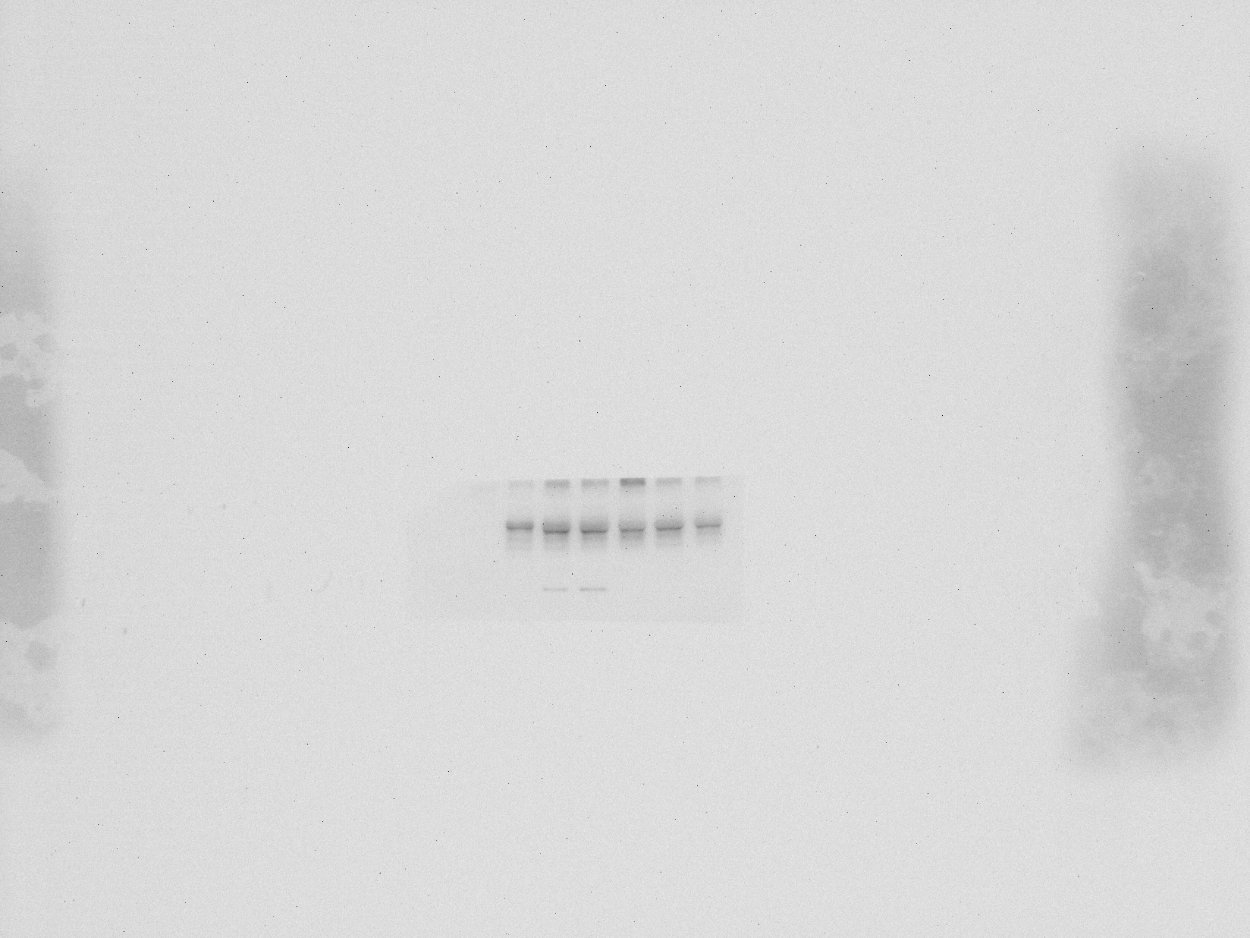


**(B) Vasohibin-2**


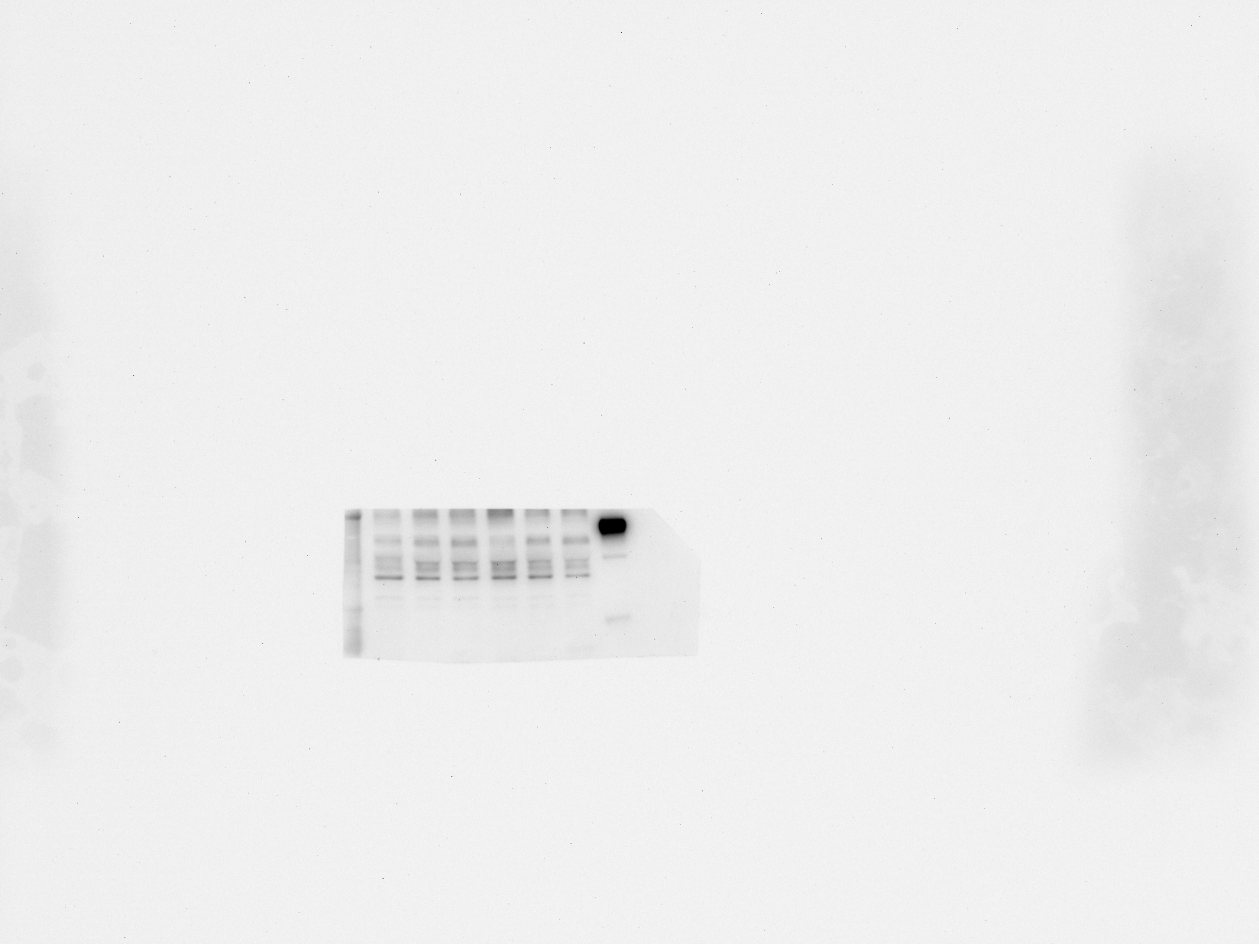


**(C) β-actin**


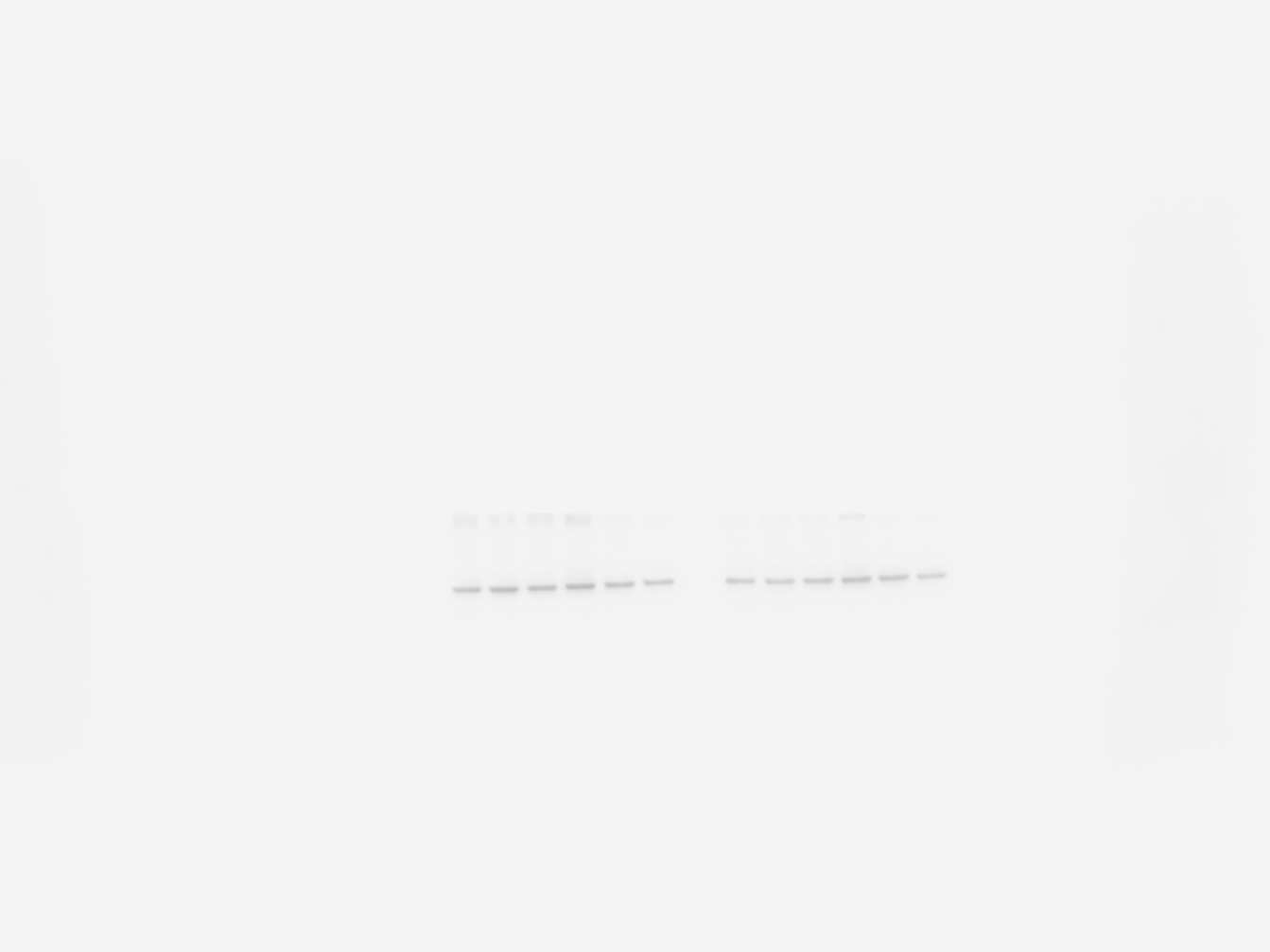


**(D) actin maker**


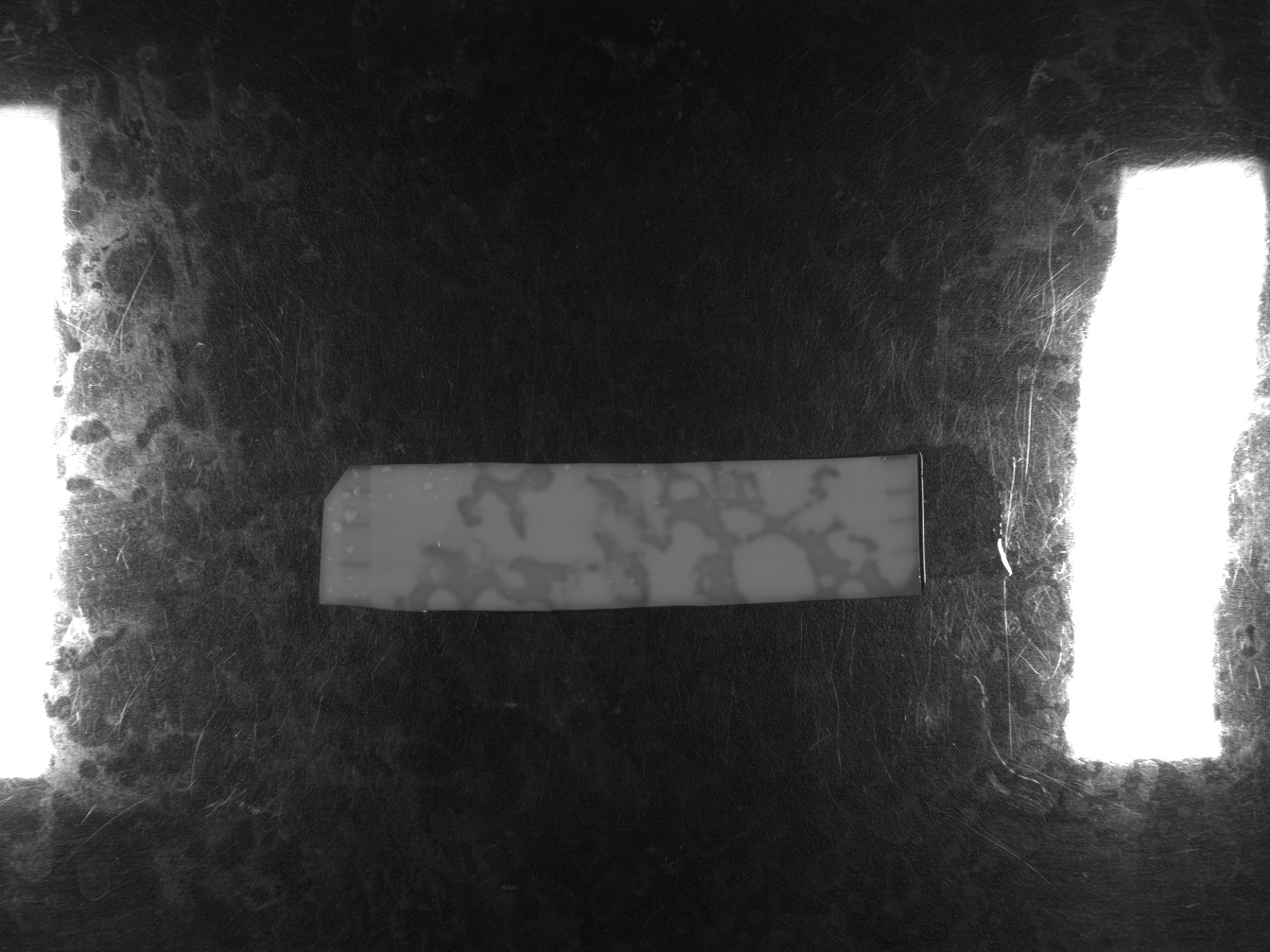


**Figure S2.** The full uncropped image of representative gels of baseline iNOS phosphorylation (p-iNOS) in WBCs (A) and p-iNOS maker (B)

**(A) iNOS phosphorylation**


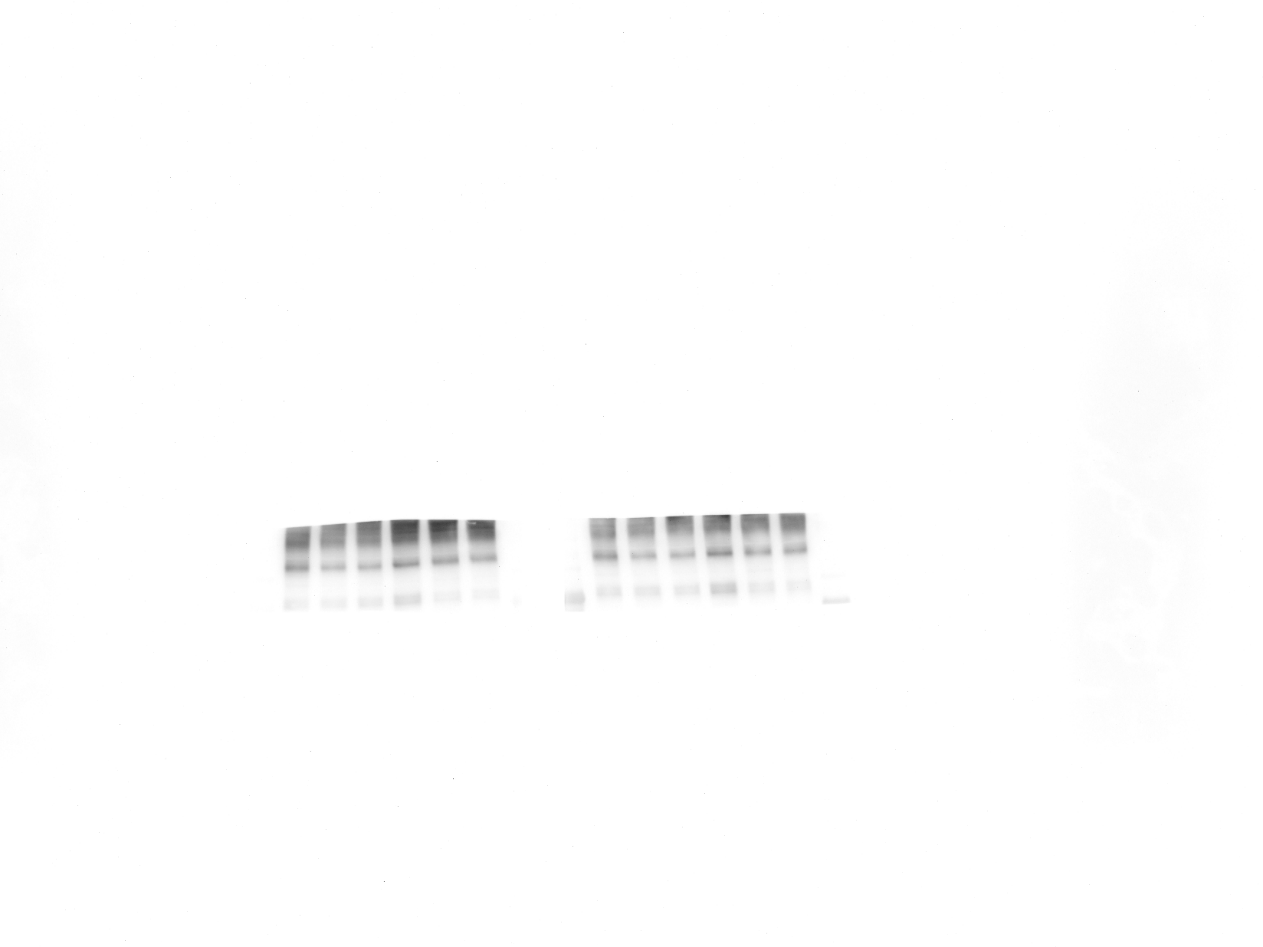


**(B) p-iNOS maker**


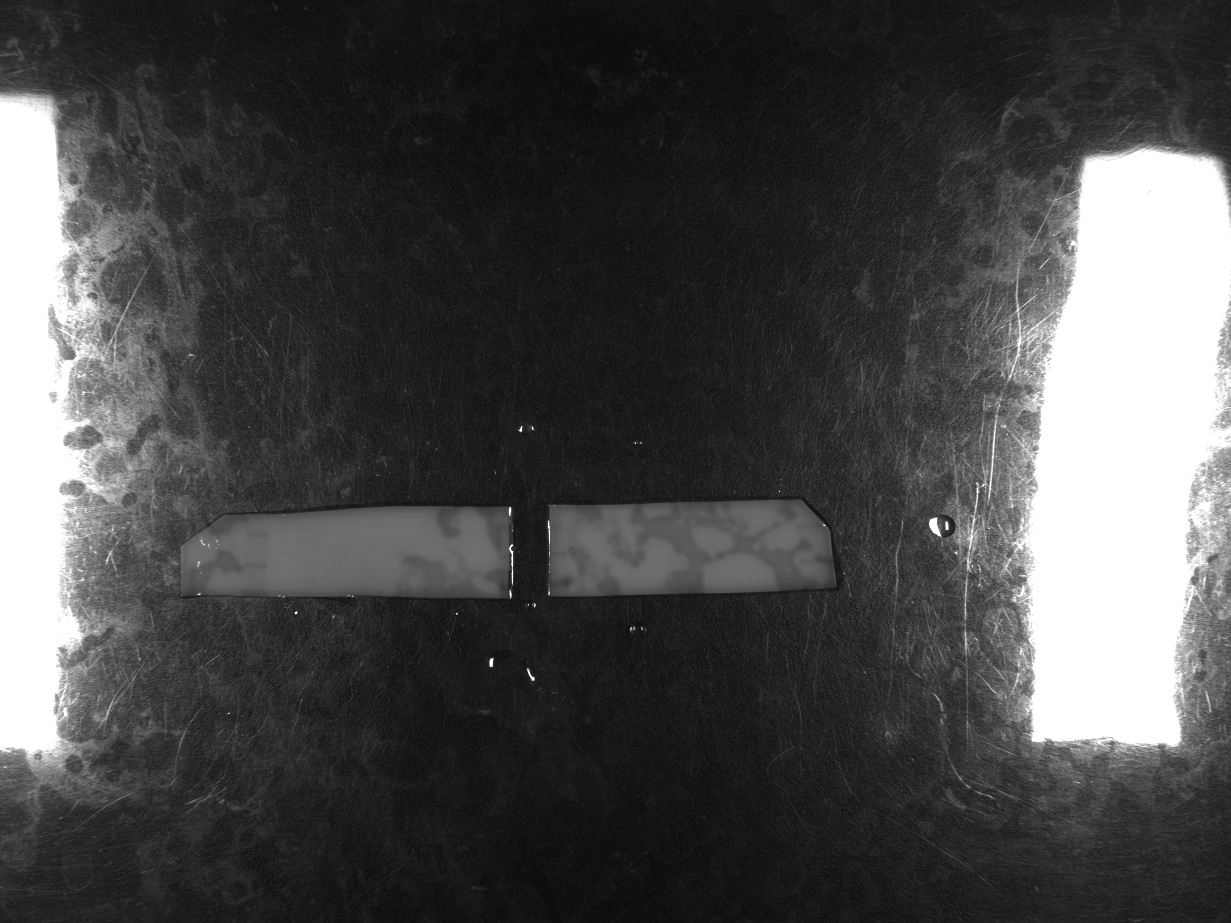


**Figure S3.** Plasma vasohibin-1 levels were significantly reduced following tumor resection in cancer patients. Data are presented as median, interquartile range, and range. (^**^*P* < 0.01, indicating a significant difference before and after surgery).

**
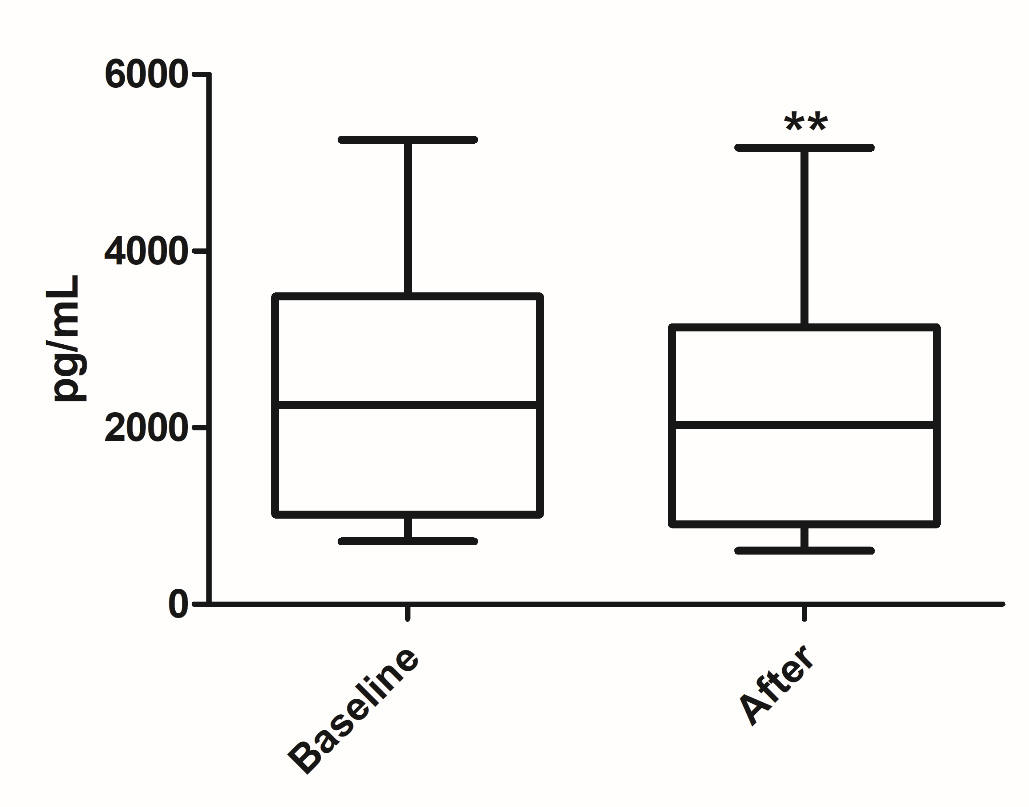
**

**Figure S4.** The full uncropped image of representative gels of vasohibin-1 (A) and vasohibin-2 expression (B) in WBCs after tumor resection and β-actin (C)

**(A) Vasohibin-1**


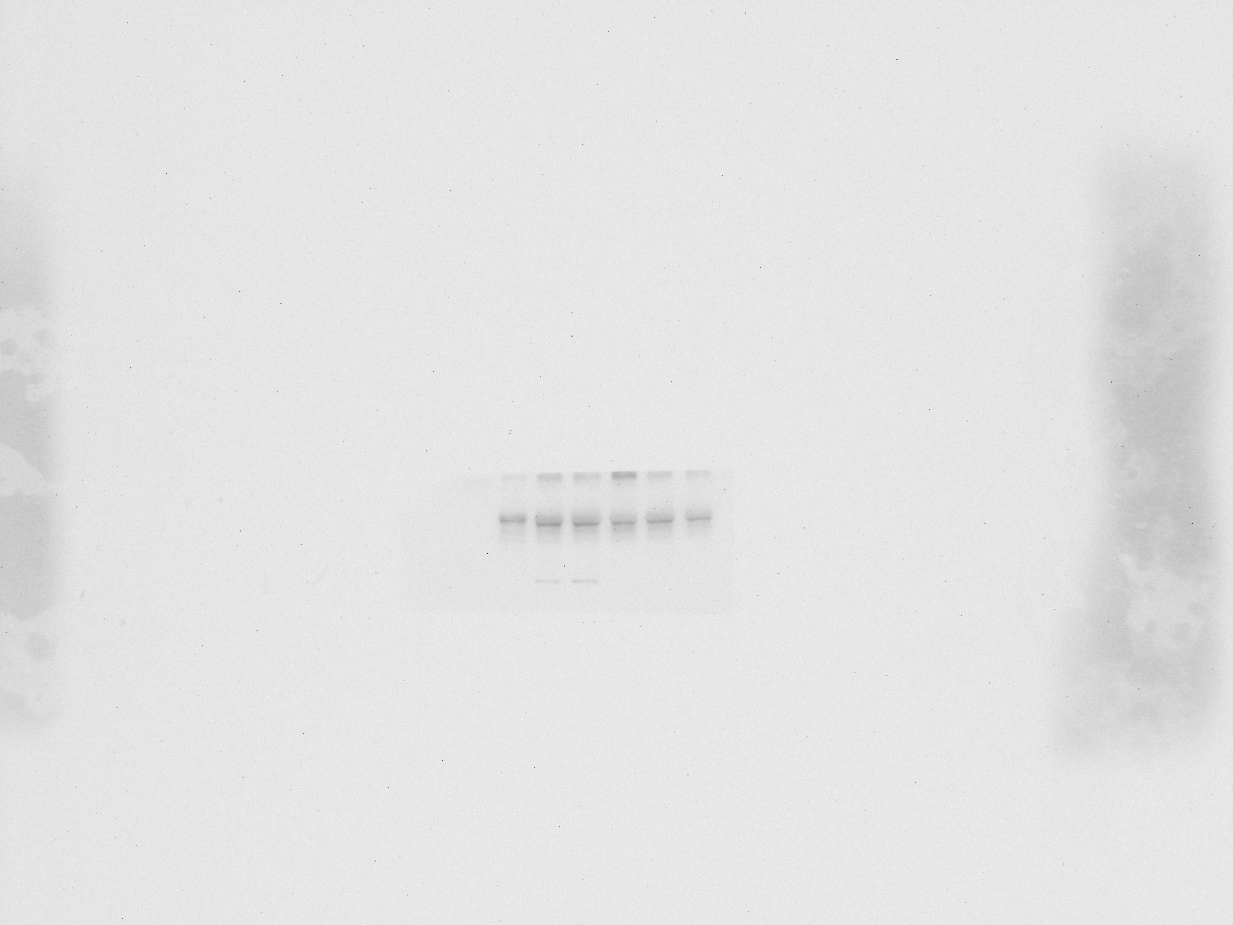


**(B) Vasohibin-2**


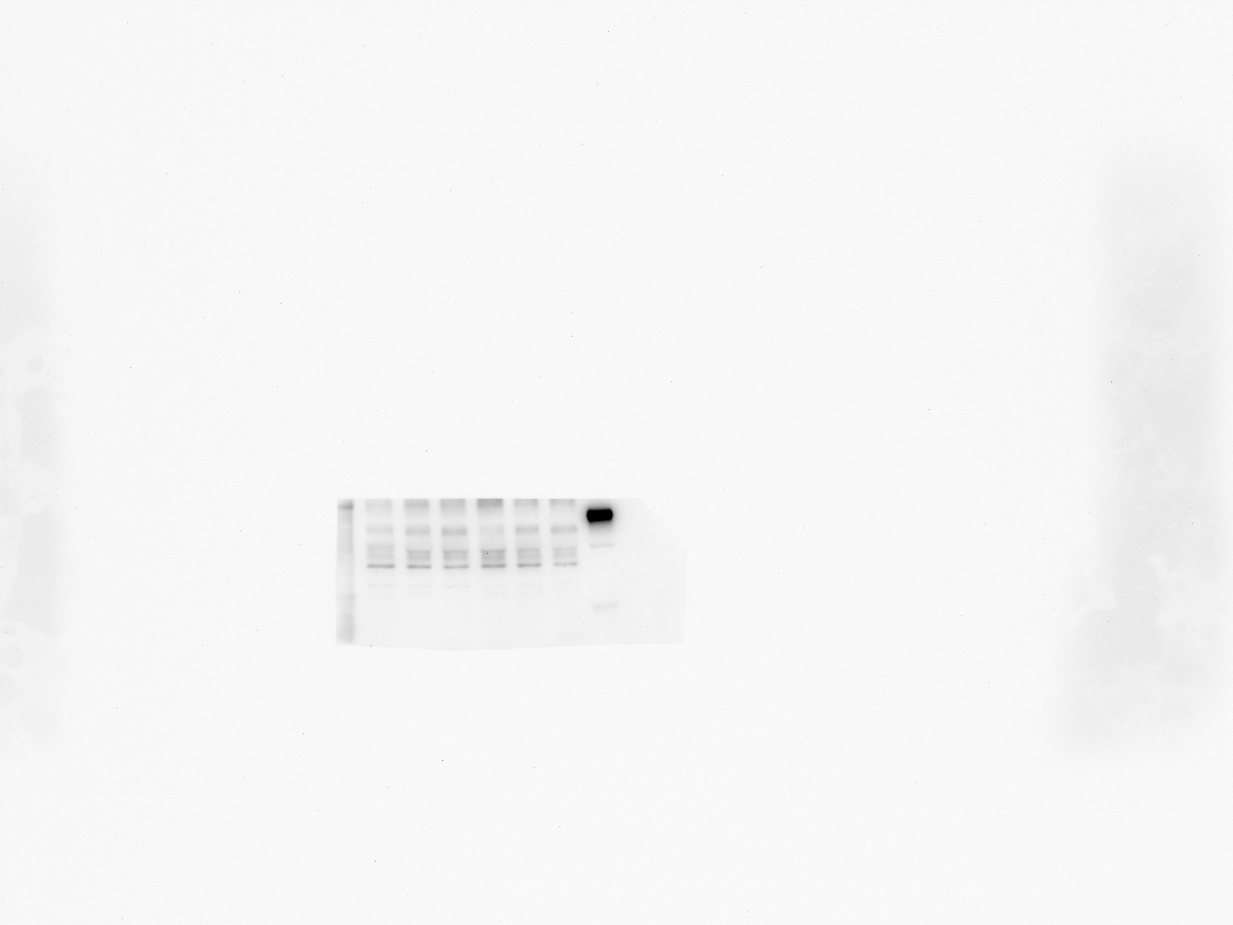


**(C) β-actin**


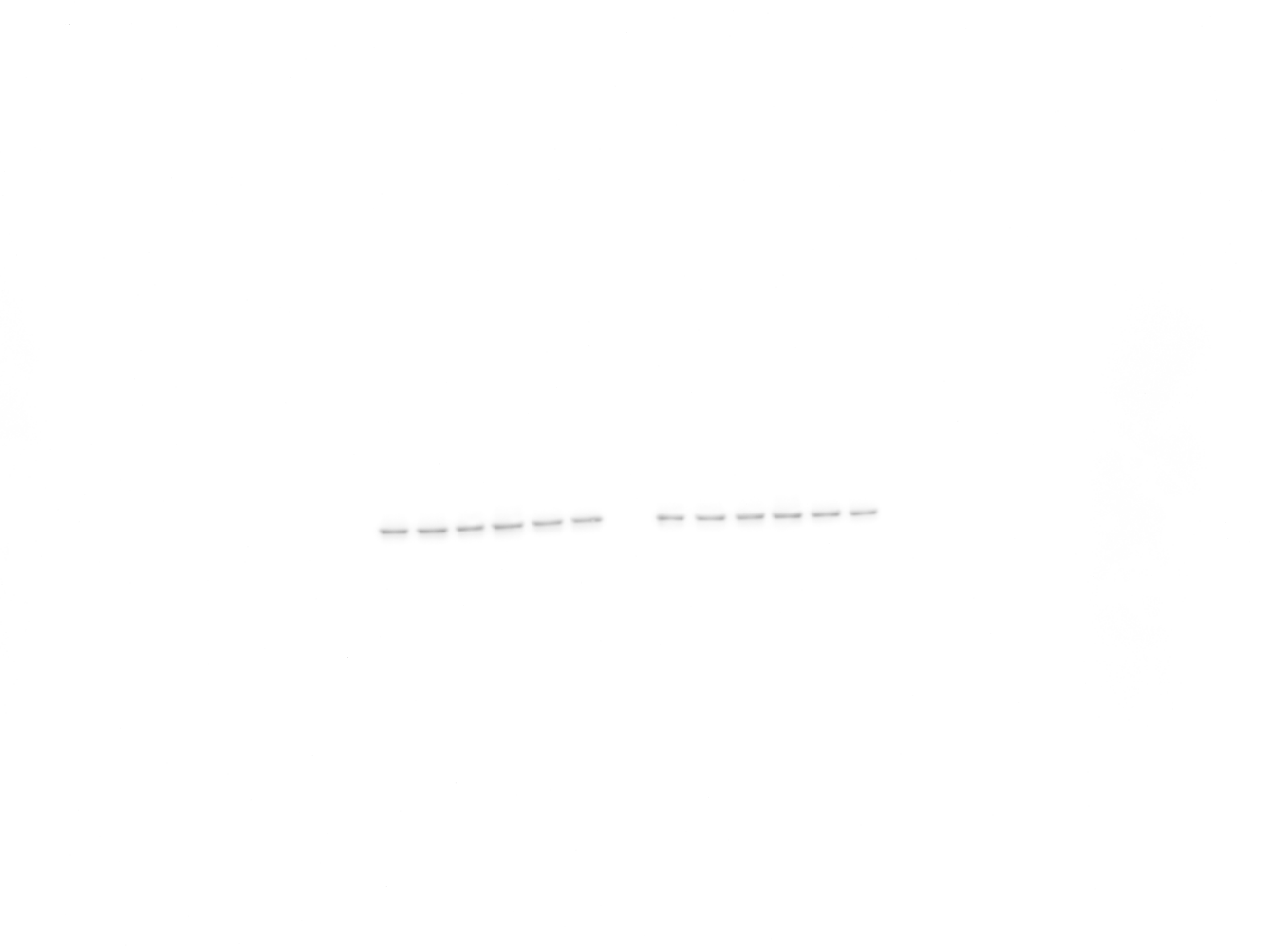


**Figure S5.** The full uncropped image of representative gels of the iNOS phosphorylation level in WBCs after tumor resection


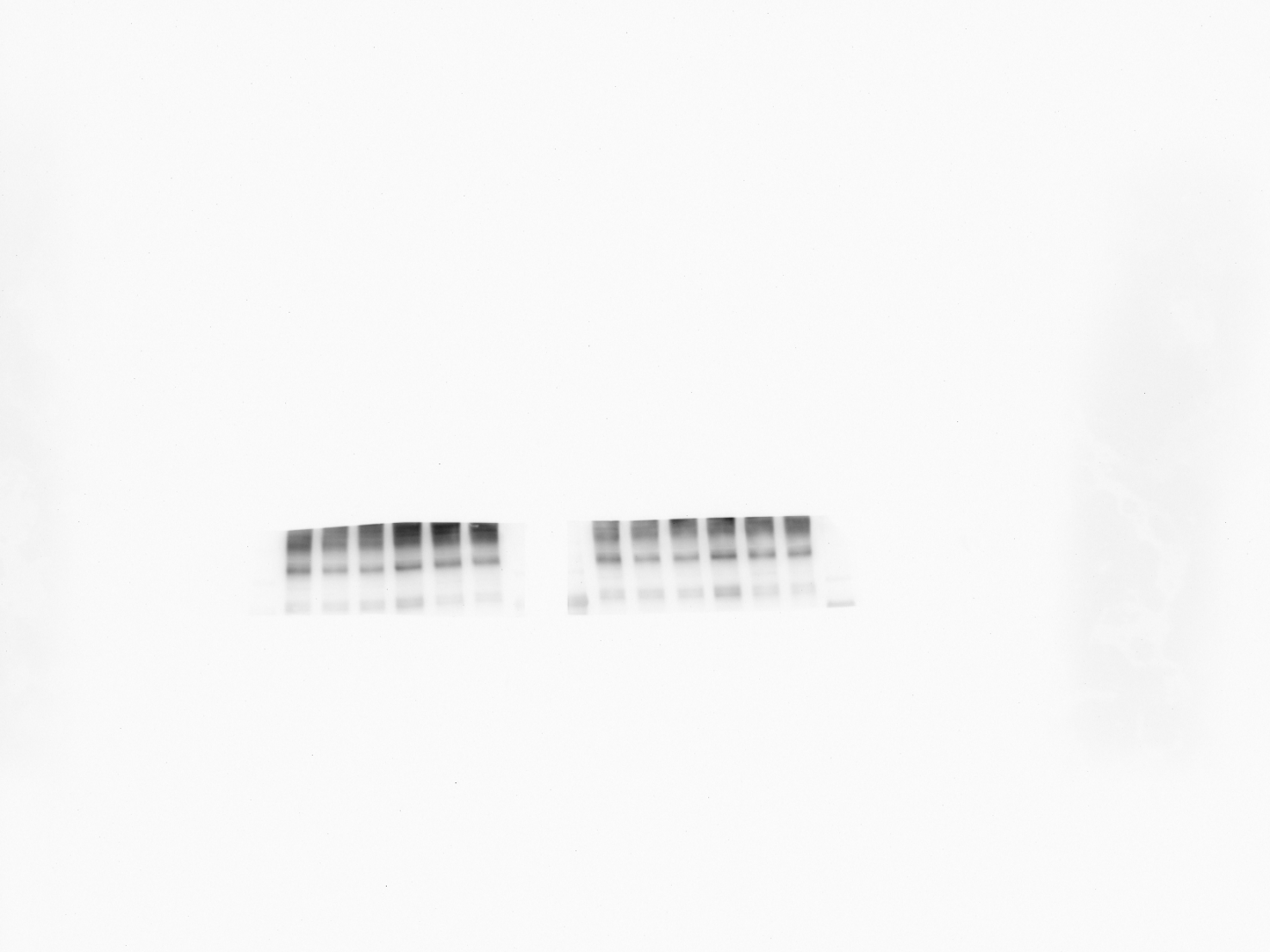

Supplement: Supplementary file 1 — Supplementary Material 1. [file 12957_2025_3853_MOESM1_ESM.docx]
